# Ammonium Inhibits Primary Root Growth by Reducing the Length of Meristem and Elongation Zone and Decreasing Elemental Expansion Rate in the Root Apex in *Arabidopsis thaliana*

**DOI:** 10.1371/journal.pone.0061031

**Published:** 2013-04-08

**Authors:** Ying Liu, Ningwei Lai, Kun Gao, Fanjun Chen, Lixing Yuan, Guohua Mi

**Affiliations:** Key Lab of Plant-Soil Interaction, MOE, Center for Resources, Environment, and Food Security, College of Resources and Environmental Sciences, China Agricultural University, Beijing, China; Wake Forest University, United States of America

## Abstract

The inhibitory effect of ammonium on primary root growth has been well documented; however the underlying physiological and molecular mechanisms are still controversial. To avoid ammonium toxicity to shoot growth, we used a vertical two-layer split plate system, in which the upper layer contained nitrate and the lower layer contained ammonium. In this way, nitrogen status was maintained and only the apical part of the root system was exposed to ammonium. Using a kinematic approach, we show here that 1 mM ammonium reduces primary root growth, decreasing both elemental expansion and cell production. Ammonium inhibits the length of elongation zone and the maximum elemental expansion rate. Ammonium also decreases the apparent length of the meristem as well as the number of dividing cells without affecting cell division rate. Moreover, ammonium reduces the number of root cap cells but appears to affect neither the status of root stem cell niche nor the distal auxin maximum at the quiescent center. Ammonium also inhibits root gravitropism and concomitantly down-regulates the expression of two pivotal auxin transporters, AUX1 and PIN2. Insofar as ammonium inhibits root growth rate in AUX1 and PIN2 loss-of-function mutants almost as strongly as in wild type, we conclude that ammonium inhibits root growth and gravitropism by largely distinct pathways.

## Introduction

Ammonium is a major component of nitrogen fertilizer and, at low concentration, it is a preferential nitrogen source and promotes growth of most plant species [1]. In many agricultural soils, ammonium is over accumulated because of the application of large quantities of urea-based fertilizer, which is further converted into ammonium by microbes [2], [3]. Typically, plants grow less vigorously on ammonium as a sole nitrogen source compared with growth on nitrate, and excess ammonium is often considered to be toxic to plants. Many hypotheses have been offered to explain ammonium toxicity, including ionic imbalance, intracellular pH disturbance, carbon imbalance, and unidirectional ammonium flux. However, none of these theories are universally accepted [4], [5].

Ammonium at a high concentration (that is, 10 mM or above) usually inhibits primary root development [5]. Recently, Qin et al. [6] found for arabidopsis plants grown on agar that an essential determinant of the sensitivity of primary roots to high concentrations of ammonium was guanosine 5′-diphosphate-mannose pyrophsphorylase (GMPase). Root growth inhibition by ammonium in arabidopsis has also been shown to be mediated by the root tip and linked with a significant ammonium efflux at the elongation zone [7]. In *Lotus japonicas,* 20 mM ammonium significantly represses root growth without affecting shoot biomass or amino acid content, as compared to treatment with 20 mM Gln [8]. These findings suggest that ammonium inhibits root growth by perceived as a signal rather than by an assimilatory negative feedback mechanism. Moreover, this inhibition of root growth can be phenocopied by over expression of a high-affinity ammonium transporter *LjAMT1;3*, and this action is independent of ammonium supply [8].

Although the inhibitory effect of ammonium on primary root growth has been previously studied by genetic or physiological approaches, the underlying mechanisms are still unclear and cause some controversy. First, in previous studies, ammonium was often supplied together with nitrate and at high concentrations (10 mM or even higher), which does not reflect the ammonium status of a typical aerobic agricultural soil where the concentration of ammonium in solution is generally less than 1 mM [2]. In arabidopsis, when plants were supplied with ammonium at 10 mM and above, shoot growth was remarkably repressed [9]. In order to avoid the combined toxic effects of high ammonium, it is necessary to investigate the mechanism to regulate root growth when the root tip is in contact with physiological concentrations of ammonium (<10 mM) as a sole nitrogen source.

Second, root growth reflects the production of cells and their expansion [10]. In evaluating root growth, one needs to distinguish the unitary processes (cell cycle vs. expansion) from the integrated output (total cells produced vs. total root length added). For clarity, we will refer here to the unit division process as “cell division” in contrast to the integrated output of cells as “cell production”. Likewise, we will use “elemental expansion” to refer to the unitary elongation process and use “root growth rate” to refer to the total output [11]. Li et al. [7] reported that final cell length was rapidly and significantly repressed by high ammonium (60 mM) while cell division was not much changed, and concluded that the reduction of cell elongation is the main influence. This conclusion was based mainly on analyzing the length of the mature cortical cells and the expression pattern of a cell cycle reporter *CycB1;1::GUS* in the root meristem. However, final cell length itself is not enough to reflect the process of elemental expansion. A kinematic analysis is necessary to quantify the influences of ammonium on both elemental expansion and cell production with more detailed root growth parameters [10], [11].

Third, the hormone auxin is an essential regulator of root development [12]. Regulatory effects of auxin on root development correlate with auxin spatial and temporal distribution and homeostasis in plant cells, which is facilitated by auxin influx transporters (AUX1), auxin efflux carriers (PINs), and auxin response factors (AXRs) [13], [14], [15], [16]. Root growth is inhibited by exogenous auxin and by auxin transport inhibitors, suggesting that root growth is sensitive to auxin levels. Cao et al. [17] showed that the auxin mutants *aux1*, *axr1*, and *axr2* are less sensitive to the inhibitory effect of ammonium on root growth. Moreover, Kudoyarova et al. [18] observed a lower auxin content in wheat roots supplied with ammonium compared to nitrate suggesting that auxin participates in ammonium inhibition. However, Li et al. [7] found that neither auxin nor ethylene participated in the ammonium inhibition of root growth as auxin-transport mutants and ethylene-insensitive mutants were still sensitive to ammonium. Zou et al. [19] find ammonium induces an agravitropic phenotype in roots, which is related to lateral auxin redistribution and largely independent of inhibitions on root elongation. From this it is clear that further work is required to fully understand the role of auxin in the ammonium induced inhibition of root growth.

In this study, the effect of external ammonium supplied at physiological concentrations on primary root growth was investigated. To avoid combined toxic effects of high ammonium on the shoot growth, we used a vertical two-layer split plate agar system. With nitrate in the upper layer, nitrogen status was maintained, and only the apical portion of the root system contacted the lower layer, which contained ammonium. Using this system, we find that strong inhibition of root growth by ammonium occurs even at 1 mM. Then, using a kinematic approach, we find that ammonium inhibits both elemental expansion and cell production. Finally, we re-evaluate the role of auxin in the regulation of ammonium on root elongation and gravitropism.

## Materials and Methods

### Plant Material, Growth Conditions, and Chemicals

All plant lines used were in the Columbia background of *Arabidopsis thaliana*. The auxin mutants *aux1-7* [20] and *eir1-1* (a loss-of-function mutant for *PIN2*) [21], the cell cycle marker line *CycB1;1::GUS* [22], and the enhancer trap lines identifying columella cells *CS31333* [23] were obtained from the Arabidopsis Biological Resource Center, Columbus Ohio, USA. Transgenic lines *AUX1::GUS* [24] were provided by Prof. B Scheres at the University of Utrecht. *PIN2-PIN2::GFP* [25] were from Prof. Jiří Friml at Ghent University. The quiescent center specific marker line *WOX5::GFP* and columella stem cells specific enhancer trap line *J2341* were provided by Prof. Chuanyou Li of the Institute of Genetics and Developmental Biology, Chinese Academy of Sciences, and are described in Blilou et al. [26].

**Figure 1:**
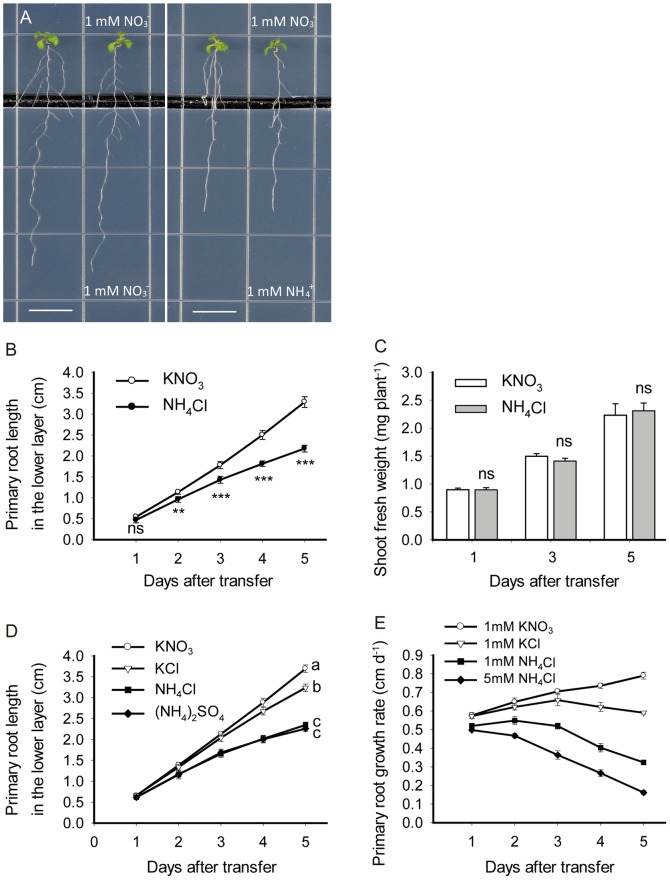
Primary root growth is inhibited by ammonium in *Arabidopsis thaliana*. *A. thaliana* Columbia plants were precultured on basal medium with 1 mM KNO_3_ as the sole nitrogen source, and then seedlings with 2 cm primary roots were transferred to a vertical two-layer split plate system with 1 mM nitrate in the upper layer and either 1 mM nitrate or ammonium in the lower layer, thus only apical portion of the root system contacted the treatment medium. (A) Photographs of representative seedlings in nitrate and ammonium treatment at five days after treatment. Bar = 1 cm. (B) Primary root length. Data are means ± SE (n = 4) from four replicate plates, with 4 seedlings each. Means for nitrate and ammonium treatment are significantly different: * p<0.05; ** p<0.01; *** p<0.001; ns = not significant. (C) Shoot fresh weight. Data are means ± SE (n = 4) from four replicate plates, with 4 seedlings each. (D) Primary root length in response to different forms of nitrogen. Data represent means ± SE (n = 4) from four replicate plates, with 4 seedlings each. Different letters indicate significant differences at p<0.05. (E) Primary root growth rate with the indicated composition of in the lower layer. Data represent means ± SE (n = 4) from four replicate plates, with 4 seedlings each.

Seeds were surface sterilized for 6 min in 8% NaClO (v/v) (in 95% ethanol), washed by anhydrous ethanol and dried. They were then placed in plates with agar growth medium and stored at 4°C in the dark for two days. The growth medium contained: 1 mM KNO_3_, 1.25 mM KH_2_PO_4_, 1.5 mM MgSO_4_, 3 mM CaCl_2_, 5 µM KI, 0.1 mM H_3_BO_3_, 0.1 mM MnSO_4_, 0.03 mM ZnSO_4_, 1 µM Na_2_MoO_4_, 100 nM CuSO_4_, 100 nM CoCl_2_, 0.1 mM Na_2_-EDTA, 0.1 mM FeSO_4_, 1% agar, and 1% sucrose. The pH was adjusted to 5.8 with KOH. Dishes were oriented vertically in a growth chamber with a 22/18°C, 16/8 h day/night regime. One week later, seedlings with 2 cm primary roots were transferred to a two layer vertical split root agar system for localized ammonium treatment (Figure 1A). The upper and the lower layers of the split-plate system were separated by a 2 mm wide trench so as to avoid leaching between two layers. For the ammonium treatment, the medium in the upper layer contained 1 mM KNO_3_ and the lower layer contained 1 mM NH_4_Cl with 1 mM KCl added to keep potassium levels invariant between the two treatments. For positive controls, the plates contained 1 mM KNO_3_ in both layers. For the non-nitrogen controls, 1 mM KNO_3_ was added to the upper layer and 1 mM KCl was added to the lower layer.

### Analysis of Root Growth

Roots in the Petri dishes were scanned every day and the image analyzed by Optimas Image Analysis software (Version 6.1, Media Cybernetics Inc., Silver Spring, MD, USA). Data were analyzed using SAS program.

In root phenotype experiments, four plates each containing four seedlings were tested. Unless otherwise indicated, three to four independent experiments were conducted for all the root phenotype assays. Data from one representative experiment were used for variation analysis using Microsoft Excel.

### Kinematic Analysis of Root Growth

Cortical cell length was determined from roots that were stained with propidium iodide (PI). Briefly, primary roots were stained in solution containing 100 µM PI for 1 min, then washed several times in distilled water, and imaged by a laser confocal scanning microscope (Eclipse TE2000-E, Nikon, Japan) with excitation and emission wavelengths of 543 and 617 nm, respectively. Final cell length [L_f_; µm] was measured by the length of cortical cells in the root maturation region where root hairs reach their maximal length on the fifth day after treatment, using the manufacturer’s software (EZ-C1 3.00, Nikon). Six to seven roots were analyzed for each treatment, and at least eight to ten mature cortical cells were measured for each root. Cell production rate [CPR; cell day^−1^] was calculated by dividing primary root growth rate by final cortical cell length for each root separately [10].

Local cortical cells length [L(x); µm] was then measured along primary root axis with the same roots used for the measurement of final cell length and cell production rate. Data of cells within the same 100 µm interval were pooled. Data of cortical cell length from each root were smoothed and interpolated to 25 µm intervals as described previously [10], and plotted as a function of distance from the quiescent center. Under steady-state growth conditions, there is a strict correspondence along the root tip between local cell length [L(x); µm] and local displacement velocity [V(x); µm h^−1^] [27], [28]. Velocity at each position along the root axis was calculated by multiplying local cell length by cell production rate for each root separately [V(x) = L(x) × CPR; µm h^−1^]. Elemental expansion rate was obtained as the local derivative of the velocity curve [r(x) = 100 × (δv/δx); % h^−1^] [10]. Based on the profile of cell length and cell production rate, the profile of velocity and elemental expansion rate was developed, and then plotted as a function of distance from the quiescent center as well.

The apparent length of the root meristem was defined as the distance between the quiescent center to the noticeably elongated cell of which local cell length is 2 times larger than the minimum cell length in cortex files, and the number of cortical cells in this region was counted [29]. Fifteen to sixteen roots were measured in each treatment.

### Gravitropic Curvature Analysis

The gravitropic dynamics of roots in response to ammonium was tested in the two-layer split plate system. The plates were reoriented by 90° and root curvature was measured over time. The gravitropic dynamics was determined on the first and the second day after the seedlings were transferred to treatment medium. The similar results were obtained and the result on the first day was presented.

### Histochemical Analysis and Microscopy

Histochemical study of β-glucuronidase (GUS) activity was determined as described by Malamy and Benfey [30]. Briefly, whole seedlings or roots were incubated in GUS reaction buffer (0.2 mM X-gluc, 0.5 mM ferricyanide, 0.5 mM ferrocyanide, 100 mM phosphate buffer, pH 7.0, and 0.1% Triton X-100) for 2 h at 37°C in darkness. Photographs were taken with an Olympus BX51 microscope. For detection of green fluorescent protein (GFP) fluorescence, roots were first stained with PI, and then imaged under confocal microscope as described above. GFP was excited by 488 nm light and observed using a detection window from 500 nm to 530 nm, and PI-stained cell walls were excited by 543 nm light and detected by 590 nm to 620 nm.

### RNA Extraction and Quantitative Real Time PCR Analysis

Total RNA was extracted from frozen root samples (20–100 mg) by TRIzol (Invitrogen) according to the manufacturer’s protocol. To prepare template cDNA, two micrograms of RQ-DNase (Promega) and digested total RNA were reverse transcribed to one strand cDNA using Moloney murine leukemia virus reverse transcriptase (Promega) and ligo dT(18) primers, according to the manufacturer’s protocol. The gene expression level was determined by quantitative real-time PCR (ABI 7500 Real-Time PCR System). The SYBR Green Realtime PCR Master Mix Kit (TOYOBO) was used for the PCR reactions. Gene specific primers for the PCR reactions were designed to have a melting temperature of ∼58–62°C and to give a PCR product between 150 and 250 bp. Expression levels of tested genes were normalized to expression levels of Ubiquitin 10 (At4g05320). The sequences of the gene specific primers (given by Steffen Vanneste) were as follows: AUX1 (forward, *AGTAGCAAATGACAACGGAACAG*; reverse, *AGAGCCACCGTGCCATAGG*); PIN2 (forward, *CCTCGCCGCACTCTTTCTTTGG*; reverse, *CCGTACATCGCCCTAAGCAATGG*); Ubiquitin10 (forward, *CTTCGTCAAGACTTTGACCG;* reverse, *CTTCTTAAGCATAACAGAGACGAG*).

## Results

### Ammonium at a Physiological Concentration Inhibits Primary Root Growth

To investigate the responses of primary roots to external ammonium, *Arabidopsis thaliana* Columbia (Col) plants were precultured on basal medium with 1 mM KNO_3_ as a sole nitrogen source, and then seedlings with 2 cm primary roots were transferred to a vertical two-layer split plate agar system (Figure 1A). When the primary roots grew over the lower layer medium containing 1 mM ammonium, root growth was significantly inhibited two days after transfer compared to that of the 1 mM nitrate control (Figure 1A and B). At five days after transfer, the length of the primary root on ammonium treatment was ∼66% of that of the nitrate control. By contrast, shoot biomass was not significantly different between the treatments (Figure 1C), suggesting the reduction in primary root growth caused by ammonium was not due to shoot growth repression.

Nitrate can modify plant root architecture, especially by stimulating lateral root elongation [31], [32]. To verify whether the inhibition of primary root growth resulted from the absence of nitrate in the lower layer, we included a no-nitrogen control, in which the lower layer contained only 1 mM KCl (Figure 1D and E). In contrast to the inhibitory effect in the ammonium medium, primary root growth in the no-nitrogen medium was slightly slower compared to that in nitrate treatment. On the fifth day, the length of the primary root grown in the no-nitrogen medium was 12% lower than that in the nitrate control, but the length of the primary root grown in the ammonium medium was 34% lower (Figure 1D). Thus, the inhibition of primary root growth was mostly due to the presence of ammonium, while the absence of nitrate played a limited role in this process. Moreover, root growth in plants treated with (NH_4_)_2_SO_4_ was inhibited to a similar extent as that in plants treated with NH_4_Cl, minimizing the possibility of chloride toxicity (Figure 1D).

Root growth rate was measured to quantify the inhibitory effect of ammonium on primary root growth. Primary root growth rate increased each day when plants grew on the nitrate medium, while it was gradually reduced by exposure of the root tip to 1 mM ammonium (Figure 1E). The primary root growth rate in the ammonium treatment was 90% of that in the nitrate treatment on day 1, 72% on day 3, and 45% on day 5. Furthermore, a stronger inhibition of root growth occurred when the external ammonium supply increased to 5 mM, with root elongation rate being only ∼20% of the nitrate control on day 5 (Figure 1E). Taken together, the supply of external ammonium at physiological concentrations inhibits primary root growth in a time and dose dependent manner.

### Inhibition of Primary Root Growth by Ammonium is Accompanied by Reductions in both Elemental Expansion and Cell Production

**Figure 2:**
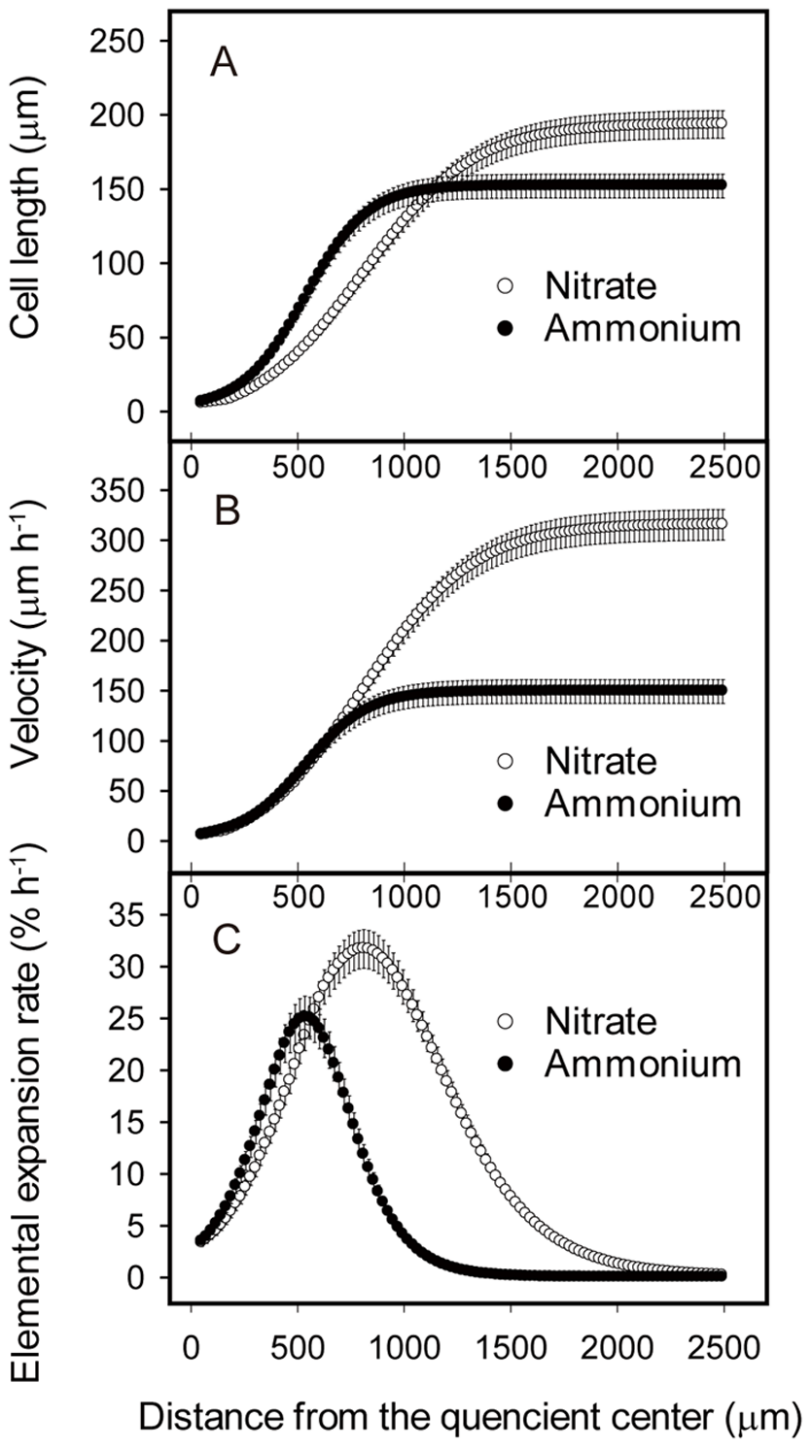
Kinematic analysis of root growth in response to ammonium treatment. Seedlings were cultured as for Figure 1A and data were obtained at five days after treatment. For (A) cell length, cortical cells were measured. Profiles of (B) velocity and (C) elemental elongation rate were obtained as described in the methods. Data plot means ± SE (n = 6 to 7).

Both elemental expansion and cell production contributes to primary root growth [10]. To determine which of these processes ammonium affects, we employed a kinematic approach to analyze root growth dynamics [10], [11]. After five days of 1 mM ammonium treatment, primary root growth rate was reduced by about one half compared to the nitrate control (Figure 1E; Table 1). We measured the spatial profile of cortical cell length and converted this profile to a velocity, the fundamental kinematic parameter, and obtained its derivative, which represents elemental expansion rate (Figure 2). Compared to the nitrate control, ammonium reduced maximal elemental elongation rate by about 22% (Figure 2C). Likewise, the ammonium treatment reduced cell production rate by about 40% (Table 1). Thus reductions in both processes of elemental expansion and cell division contribute to the inhibition of root growth caused by 1 mM ammonium.

**Table 1:** Effects of ammonium on primary root growth.

	Treatment	Root growth rate (cm day^−1^ )	Final cell length (µm )	Cell production rate (cell day^−1^ )
wild type	Nitrate	0.77±0.01 (100%)	196±8.4 (100%)	39.1±0.9 (100%)
	Ammonium	0.36±0.01 (47%)	152±7.0 (77%)	23.6±1.0 (60%)
*aux1-7*	Nitrate	0.65±0.03 (100%)	180±8.6 (100%)	35.6±1.3 (100%)
	Ammonium	0.40±0.03 (62%)	143±6.9 (79%)	28.2±1.7 (79%)
*eir1-1*	Nitrate	0.72±0. 02 (100%)	190±9.8 (100%)	37.6±1.3(100%)
	Ammonium	0.43±0.03 (60%)	143±7.2 (75%)	30.1±1.1 (80%)

Root growth parameters were analyzed kinematically at five days after treatment, as described in the methods. Values in parentheses are the percentage of control. Data represents as means ± SE (n = 6–7).

Kinematic analysis also reveals certain other features of the inhibitory effect of ammonium on root growth (Figure 2). The position, where cells exited from the meristem and began to elongate rapidly, was closer to the root apex on ammonium compared to that on nitrate. Similarly, the position where cortical cells reached their mature length was closer to the apex on ammonium compared to nitrate (Figure 2A). In addition, final cortical cell length on ammonium was reduced by ∼23% compared to that on nitrate control (Figure 2A).

The profile of elemental expansion rate on ammonium treatment not only had a lower maximum but also was truncated apically (Figure 2C). In nitrate control roots, the length of elongation zone, defined as the distance between the quiescent center and the position where cell expansion rate approached zero [10], was ∼2000 µm, and maximum elemental expansion rate was 32% h^−1^. By contrast, in ammonium treated roots, the length of the elongation zone was reduced to ∼1200 µm and maximum elemental expansion rate was 25% h^−1^, respectively (Figure 2C). Note that ammonium seems not to affect the elemental expansion rate in the apical 500 µm of primary root tips (Figure 2C). Thus, the repression of elemental expansion caused by ammonium is reflected as reductions on both the length of elongation zone and the rate of maximum elemental expansion.

### Ammonium Represses Root Cell Production by Decreasing the Size of Meristem as Well as the Number of Dividing Cells without Affecting Cell Division Rate

**Figure 3:**
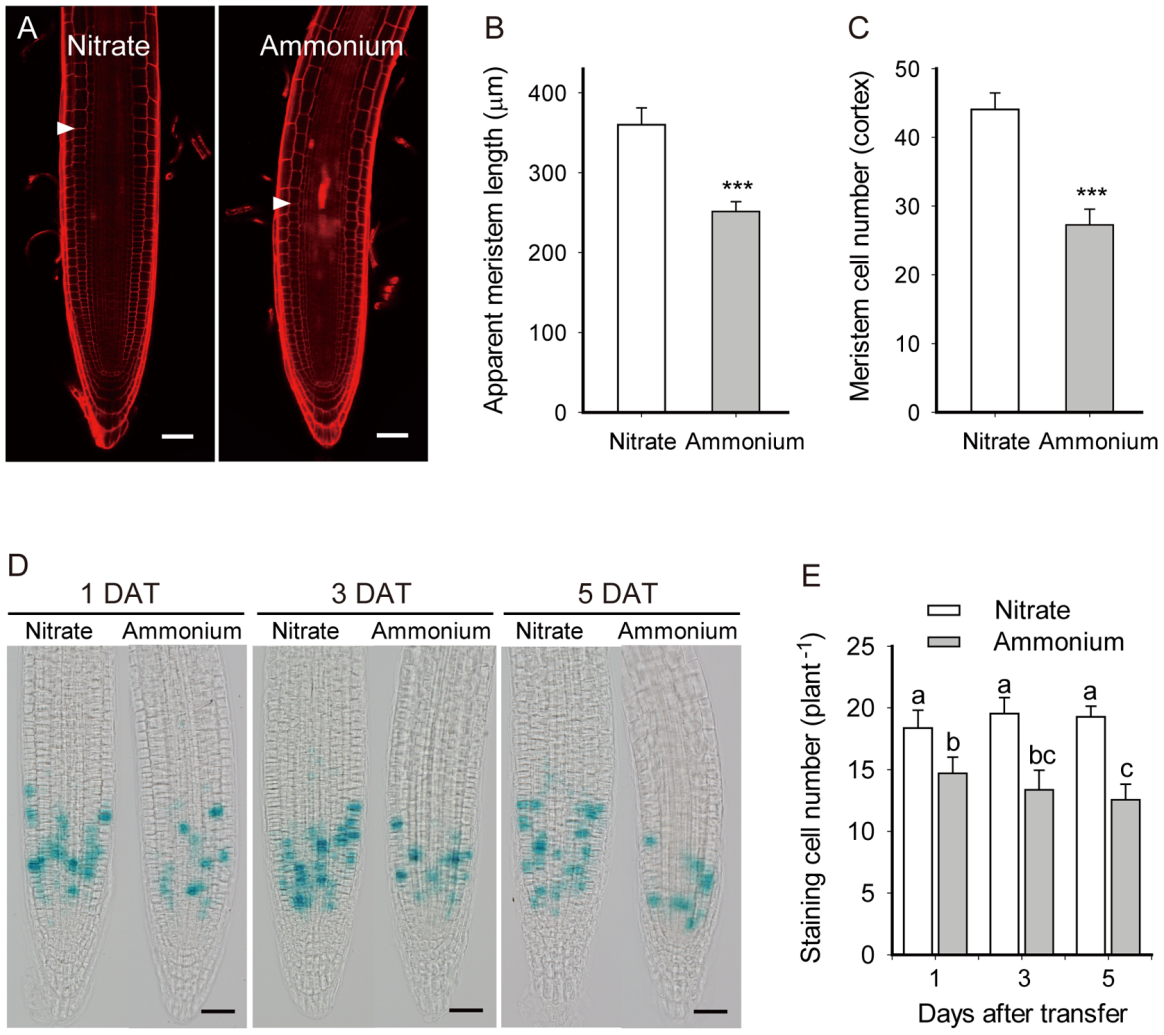
Ammonium decreases apparent root meristem size and the number of dividing cells. (A) Micrographs of PI-stained primary root tips at five days after treatment. Images are representative of 16 to 20 plants in four replicate experiments, treated as in Figure 1A. White arrowheads indicate the approximate position where cells begin to elongate noticeably. Bar = 50 µm. (B) Apparent root meristem length and (C) number of meristematic cortex cells (per file), seedlings treated as in Figure 1A. Bars plot means ± SD for 15 to 16 plants obtained in four replicate experiments. *** p<0.001. (D) Micrographs of the cell cycle reporter *CycB1;1::GUS* taken at the indicated days after transfer (DAT). Plants were treated as in Figure 1A. Bar = 50 µm. (E) The number of dividing cells as determined by counting the blue-green puncta in *CycB1;1::GUS* stained root tips. Bars plot means ± SD for 12 to 16 plants obtained in four replicate experiments. Different letters indicate significant differences at p<0.05.

Cell production depends on the number of dividing cells (i.e. the length of root meristem) and on the rate of cell division [11]. First, the approximate length of root meristem, defined as the distance between the quiescent center and the noticeably elongated cortical cell of which local cell length is two times larger than the minimum, was investigated. On day 5, the apparent length of the meristem was reduced by ∼30% by ammonium, a highly significant reduction (Figure 3B). Likewise, cortical cell number in the meristem was about 38% lower in ammonium than that in nitrate treatment (Figure 3C). These results are consistent with the reduced cell production rate under ammonium being caused partly by a reduced supply of cells.

The effect of ammonium on cell division activity was further investigated by analyzing the expression pattern of the cell cycle reporter *CycB1;1::GUS* in which glucuronidase is present mainly during the M phase [22]. Counting the approximate number of GUS-stained cells revealed that ammonium significantly reduced the number of cells in M phase with time (Figure 3D). The reduction became more pronounced in long term treatment. By day 5, the abundance of GUS stained cells was reduced by a similar extent as meristem cell number (Figure 3E). Insofar as 1 mM ammonium reduced the apparent number of dividing cells by about the same amount as the rate of cell production, we conclude that ammonium had little if any effect on cell division rate.

### Ammonium has no Apparent Effect on the Root Stem Cell Niche but Affects the Structure of the Root Cap

**Figure 4:**
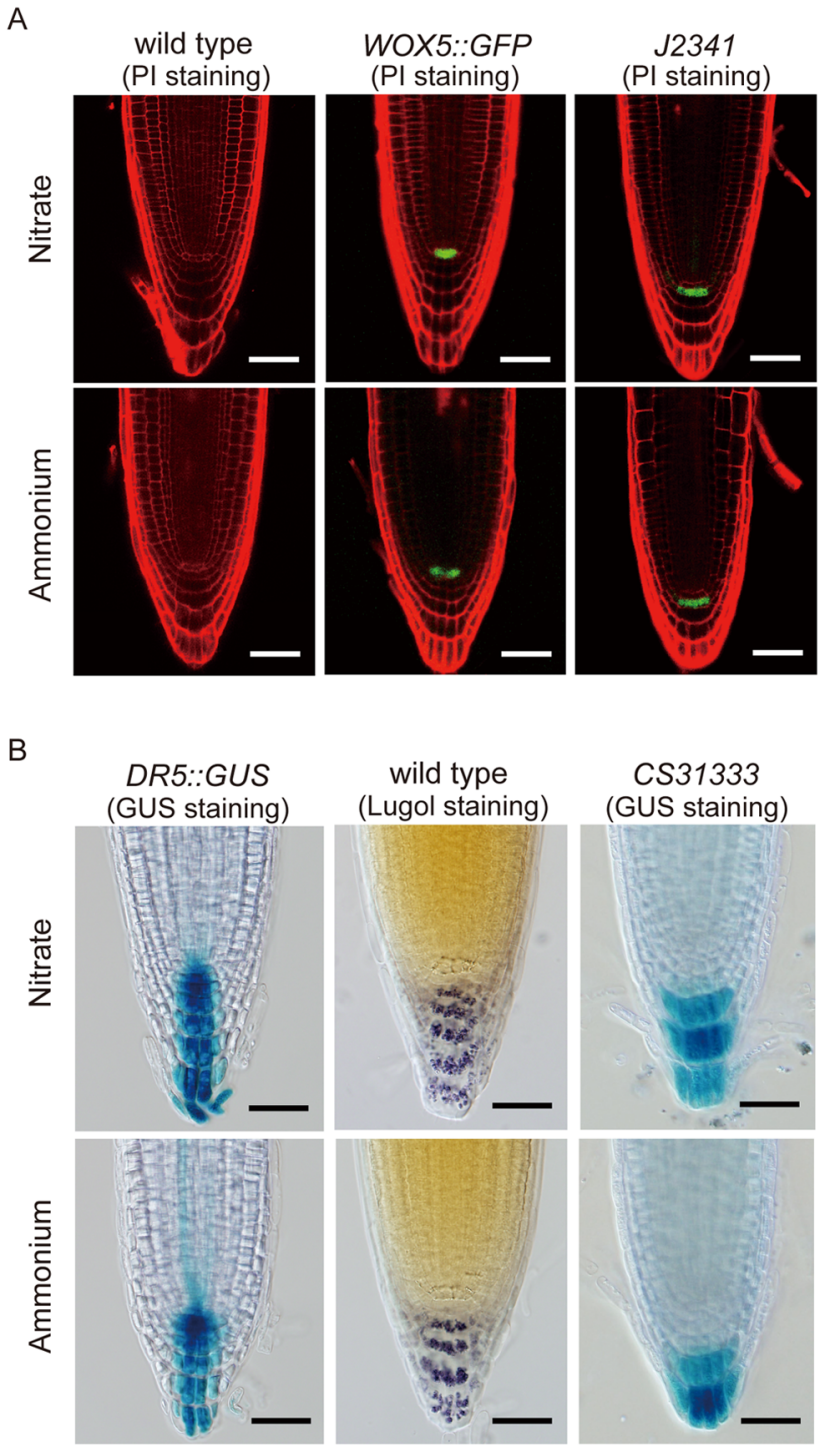
Ammonium does not alter the root stem cell activity, but reduced columella cell number. (A) Confocal fluorescence micrographs of PI-stained root tips taken at five days after treatment. *WOX5::GFP* marks the quiescent center specific marker; *J2341* marks columella stem cell. Images are representative of 12–16 plants in four replicate experiments treated as in Figure 1A. Bar = 50 µm. (B) Bright-field micrographs taken at five days after treatment. *DR5::GUS* marks response to endogenous auxin; Lugol staining marks amyloplasts; and CS31333 marks mature columella cells. Images are representative of 12–16 plants in four replicate experiments treated as in Figure 1A. Bar = 50 µm.

Root stem cells serve as the source for new derivative cells and are essential for root meristem development [12], [33]. To assess whether ammonium affected root stem cells, the cellular organization of the stem cell niche was investigated. On day 5, cellular organization of the quiescent center was apparently not affected by ammonium, as assessed by either PI staining or the quiescent center specific marker *WOX5::GFP* [19] (Figure 4A). Similarly, columella stem cells showed few if any aberrations, as evaluated by several markers, including the enhancer-trap line J2341, which expresses specifically in columella stem cells (Figure 4A), the auxin maximum as revealed by *DR5::GUS* (Figure 4B), and amyloplast development as revealed by Lugol staining (Figure 4B). Evidently, ammonium has little if any impact on the root stem cell niche.

However, ammonium appeared to reduce the number of cell tiers within the columella. This was apparent not only with Lugol staining but also with the enhancer trap line CS31333, which identifies mature columella cells [23] (Figure 4B). Therefore, ammonium appeared to affect the structure of the root cap modestly, perhaps by reducing the rate of production of columella cells in parallel to its reduction of meristem cells.

### Ammonium Inhibits Root Gravitropism and Concomitantly Down Regulates the Expression of AUX1 and PIN2

**Figure 5:**
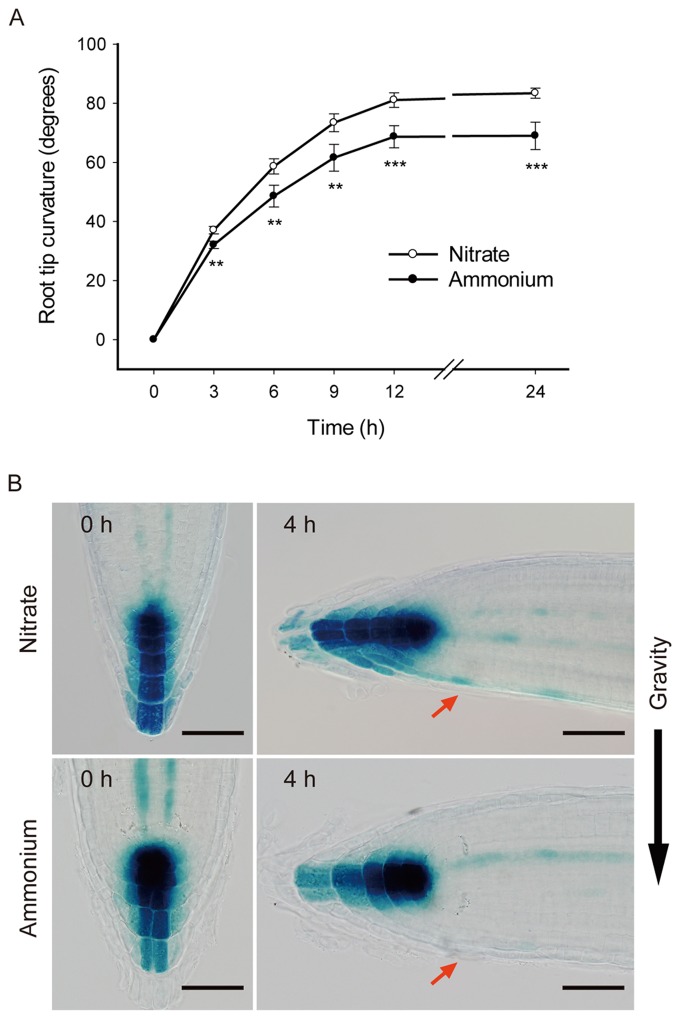
Ammonium reduces the gravitropic response of primary roots. (A) Primary root curvature in response to gravistimulation. Seedlings were cultured as described in Figure 1A. One day after transferred to 2-layer split plate medium, the seedlings were rotated 90° and root curvature was measured over time. Data represent means ± SD for 16 plants obtained in four replicate experiments. Means for nitrate and ammonium treatment are significantly different: ** p<0.01; *** p<0.001. (B) *DR5::GUS* expression pattern in root cap in response to gravistimulation. *DR5::GUS* plants were cultured as described in Figure 1A. Five days after transfer, the seedlings were rotated 90°. Plants were stained with GUS reaction buffer at the indicated times. Red arrow indicates the lower side of the root apex. Images are representative of 10–12 stained plants in three replicate experiments. Scale bars = 50 µm.

The gravitropic dynamics of roots in response to ammonium was tested in the two-layer split plate system. Compared to nitrate controls, the gravitropic response of roots on 1 mM ammonium was compromised; both the rate of bending and the final angle were reduced (Figure 5A). Evidently, ammonium interferes with some aspect of gravitropic perception or response.

Gravity-induced auxin redistribution in the root apex is shown to be essential for plants to perceive the gravitropic signal [34]. Thus, we monitored *DR5::GUS* expression patterns after a short term gravistimulation. On day 5 of treatment, vertically growing plants had a symmetric auxin distribution pattern in the root apex on either nitrogen source (Figure 5B). Four hours after reorientation by 90°, the nitrate control root tips presented the typical asymmetric *DR5::GUS* expression pattern associated with gravitropism, namely enhanced GUS expression on the lower side of lateral root cap and epidermis extending into the elongation zone (Figure 5B). However, in ammonium treated plants, the distribution pattern of *DR5::GUS* was still symmetrical (Figure 5B), suggesting that the auxin redistribution (from root apex to base) in response to gravistimulation was affected by ammonium.

**Figure 6:**
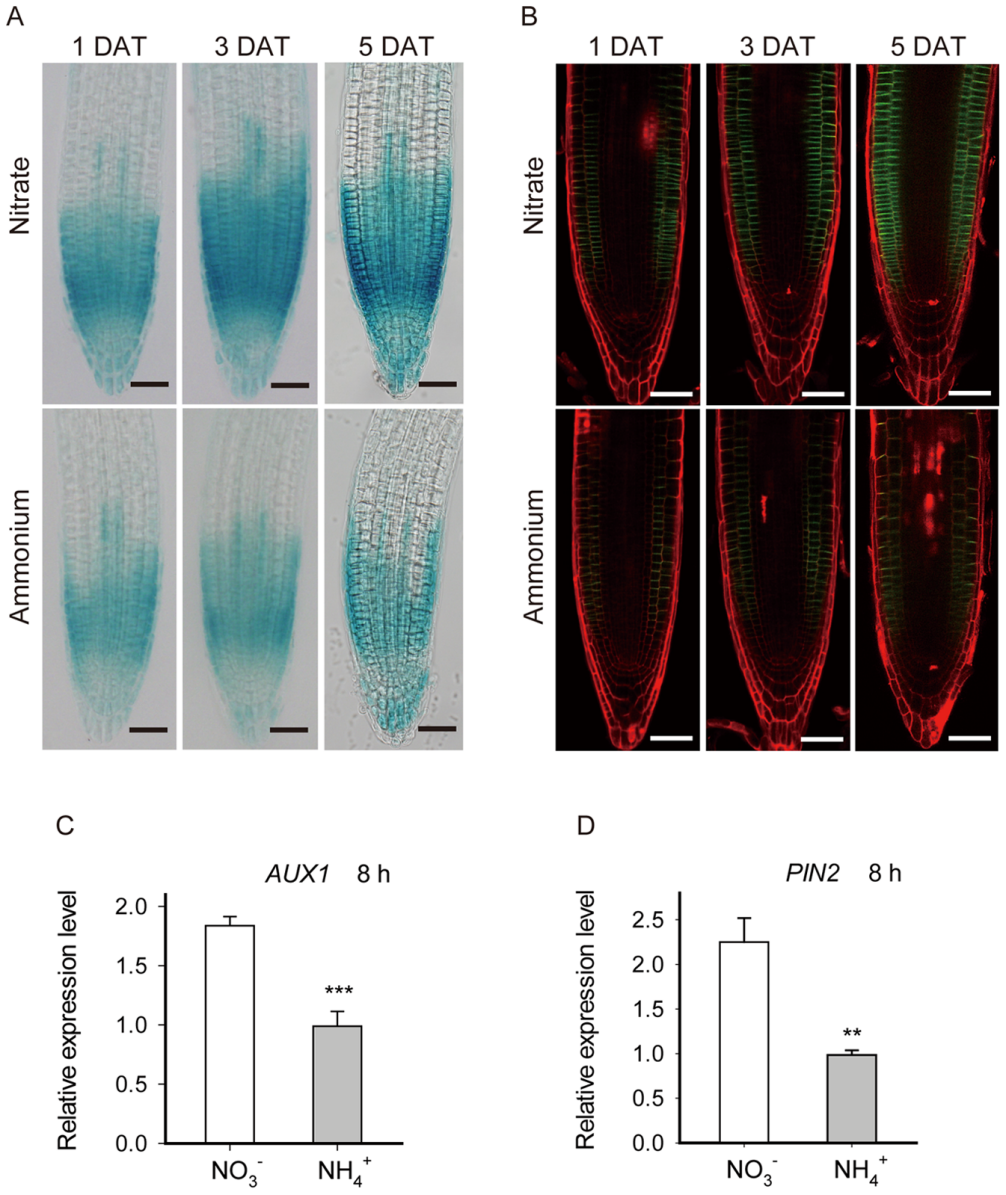
Expression of AUX1 and PIN2 is down-regulated in response to ammonium treatment. (A) *AUX1::GUS*. Bright-field images of root tips are representative of 10–12 plants in three replicate experiments, treated as for Figure 1A, and taken at the indicated days after transfer (DAT). Bar = 50 µm. (B) *PIN2::PIN2-GFP.* Confocal fluorescence images of PI-stained root tips, representative of 9–12 plants in three replicate experiments. Seedlings were treated as in Figure 1A, and tested at the indicated days after transfer (DAT). Bar = 50 µm. (C) and (D), Relative gene expression level of (C) *AUX1* and (D) *PIN2* was determined by quantitative real-time PCR. Plants were treated as in Figure 1A. The apical 5 mm segments of primary roots were sampled 8 h after transfer. At least 102 to 108 seedlings from three replicate experiments were collected for total RNA extraction. Bars plot means ± SD.

To investigate how ammonium affected auxin distribution at the root apex, we assayed well documented reporter lines relating to auxin transport, including *AUX1::GUS, PIN1::PIN1-GFP*, *PIN2::PIN2-GFP*, *PIN3::PIN3-GFP*, *PIN4::PIN4-GFP*, and *PIN7::PIN7-GFP* [13], [14], [15], [16], [35], [36]. Ammonium treatment appeared to strongly down-regulate the expression of *AUX1::GUS* and *PIN2::PIN2-GFP* (Figure 6A and 6B). The expression of *PIN3::PIN3-GFP* and *PIN7::PIN7-GFP* was slightly down regulated while the expression of other PIN::GFPs was scarcely affected (data not shown). Therefore, AUX1 and PIN2 are likely to be involved in the regulation of auxin redistribution in response to ammonium. The expression of *AUX1* and *PIN2* at the transcriptional level was quantified at 8 h after ammonium treatment by means of quantitative real-time PCR (Figure 6C and 6D). These real-time PCR data confirm that ammonium down regulates the expression of AUX1 and PIN2.

### Ammonium Inhibits Primary Root Growth in aux1-7 and eir1-1 Mutants

**Figure 7:**
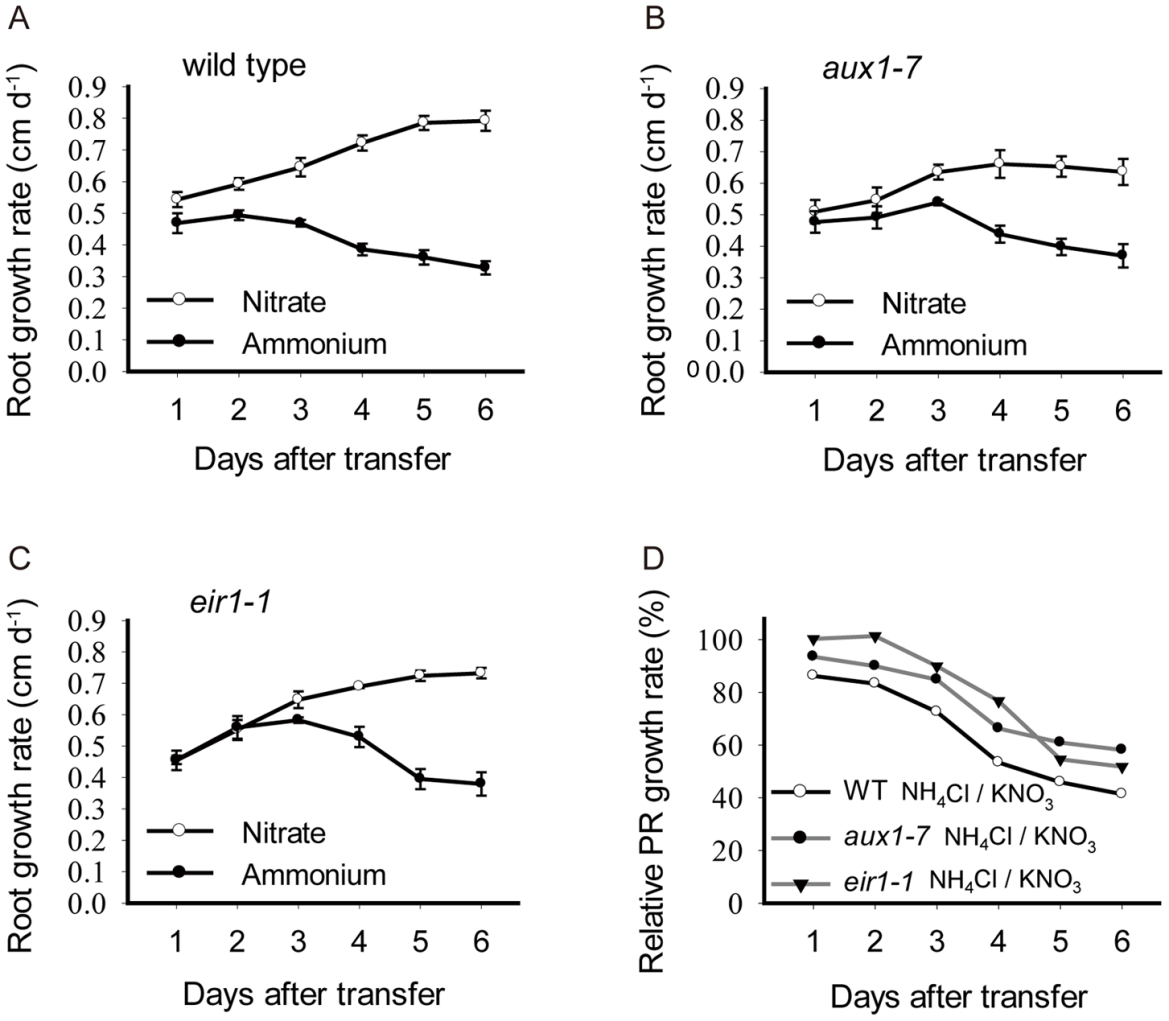
The effect of ammonium on primary roots growth in *aux1-7* and *eir1-1* mutants. (A) to (C), Primary root growth rates for (A) wild type, (B) *aux1-7*, and (C) *eir1-1*. Plants were treated as in Figure 1A. Data represent means ± SE (n = 4) from four replicate plates, with 4 seedlings each. (D) Relative primary root growth rates for the indicated genotypes. The data of root growth rates were re-plotted as a percent of plants treated with 1 mM nitrate respectively.

To determine to what extent decreased expression of AUX1 and PIN2 mediates the effect of ammonium on root growth, we assayed the growth responses of *aux1-7* and *eir1-1* (a loss-of-function mutant for *PIN2*) [21]. If ammonium inhibits root growth by down regulating AUX1 and PIN2, then root growth of the mutants should phenocopy ammonium treatment even on nitrate, and root growth in the mutants should be relatively unresponsive to ammonium. However, there was little if any difference in root growth rate between the wild type and these mutants when grown on uniform nitrate plates (Figure 7A to 7C). Moreover, both mutants responded to ammonium similarly to the wild type, although the mutants were slightly less responsive (Figure 7A to 7D). Mature cell length and cell production rate of these mutants were investigated, and these were also inhibited by ammonium in the mutants almost as strongly as in wild type (Table 1). These results suggest that ammonium decreases root elemental expansion and cell production by a pathway that is largely independent of auxin transport, although the auxin status of the root might fine-tune the response. However, since ABCB4, which is an ATP-Binding-Cassette (ABC) transporter, also mediates rootward auxin transport and regulates root gravitropic response [37], we cannot rule out the role of rootward auxin transport mediated by ABCB4.

## Discussion

### Ammonium Treatment Inhibits Both Cell Production and Elemental Expansion

High concentrations of ammonium supplied as an exclusive nitrogen source can cause toxicity symptoms in many plant species and suppresses root and shoot growth [5]. As the effects of high ammonium on plant physiology are pleiotropic, it is difficult to investigate the molecular mechanism of primary root growth suppressed by external ammonium [8]. In previous studies, ammonium was supplied at a high concentration (10 mM or even higher) [6], [7], [19]. To avoid toxic effects of high ammonium, we developed a vertical two-layer split plate system. With this system, ammonium was supplied solely to the apical part of the root system at a more or less physiological concentration (1 mM), while the rest of the root system was supplied with 1 mM nitrate. The mechanisms whereby ammonium affects root growth could then be investigated without complications from toxicity or nitrogen deficiency.

Root growth is governed by two linked processes, the production of cells and expansion [10]. Walch-Liu et al. [38] found that leaf growth of ammonium treated tobacco was limited by both reduced cell number and cell expansion. Recently, Li et al. [7] claimed that high ammonium inhibited primary root growth mainly by decreasing cell length rather than cell division. But, their study did not quantify rates of cell production or elemental expansion. We show here that ammonium at a physiological concentration inhibits primary root growth in a time and dose dependent manner, and represses rates of both cell production and elemental expansion.

Cell production contributes to root growth by providing new cells as raw materials for expansion [11]. Our result shows that the primary root growth rate gradually reduced when root tips were exposed to ammonium (Figure 1E and Figure 7A). Consistently, ammonium decreased the number of dividing cells with time, and this effect becomes more pronounced in the long term response (Figure 3D and 3E). After five days of ammonium treatment, cell production rate was reduced by 40% (Table 1), thereby limiting the supply of cells to the elongation zone. It is parsimonious to view the limitation of cells moving into the elongation zone as limiting the number of elongating cells and hence the length of the elongation zone and magnitude of root growth rate [10]. Although the length of the elongation zone can be influenced by factors independent of cell production rate, the flux of cells into the elongation zone certainly plays a significant role in regulating root growth [11]. Thus, these results indicate that the decrease in cell production is a notable consequence of the root growth in response to ammonium.

In addition, the inhibitory effect of ammonium on cell production is mainly reflected as reductions in the length of root meristem and the number of dividing cells, whereas ammonium appears to affect neither the cell division rate nor the status of root stem cell niche. Similarly, high salt decreases the number of dividing cells as well as meristem length without affecting cell division rate in *A. thaliana* [39]. Interestingly, high salt does decrease cell division rate, but the decrease is only transient, suggesting that maintaining a constant cell division rate is an adaptation to environmental stress [39]. Be that as it may, changes in the number of dividing cells (i.e. the length of meristem) are observed commonly but changes in cell division rate rarely [11].

Kinematic analysis reveals that ammonium inhibits elemental expansion, reducing both the length of elongation zone and the maximum elemental expansion rate (Figure 2C). Several studies have shown that elemental expansion is repressed by environmental stresses. Under water-deficit stress, the profile of elemental expansion rate is truncated apically and narrowed in both maize [40] and soybean roots [41]. Likewise, a similar inhibition of elemental expansion is observed in low phosphorus stress in arabidopsis roots [42]. Excess sodium treatment also causes a reduction in the length of elongation zone, but the maximal elemental expansion rate is not significantly affected [39]. Although the inhibition of root growth in response to various environmental stresses is reported frequently, the underlying mechanisms are still unclear.

### Auxin Transport Facilitated by AUX1 and PIN2 Plays a Limited Role in the Process of Ammonium Inhibited Primary Root Growth

Auxin, as a pivotal plant hormone, can mediate various environmental stimuli to regulate root development [12]. Nutrient signals can be integrated into changes in auxin response through their effects on auxin perception and auxin distribution. Phosphorus deficiency enhances auxin sensitivity by increasing the expression of the auxin receptor TIR1 in roots, which results in lateral root proliferation [43]. Localized iron accumulation promotes lateral root elongation by up-regulating the auxin influx facilitator AUX1 and accumulating auxin in lateral root apices [44]. Recently, it has been shown that high ammonium (10 mM or even higher) inhibits root gravitropism and delays lateral auxin redistribution in the root apex [19], indicating a connection between the auxin pathway and ammonium treatment.

Here, ammonium supplied to the root tips affected neither the auxin maximum in the quiescent center nor the stem cell niche (Figure 4). However, after four hours of gravistimulation, 1 mM ammonium markedly reduced the asymmetric distribution of auxin in the root apex (Figure 5B). Although ammonium could be affecting gravity perception, lateral redistribution of auxin, or shootward polar transport, our data implicate the latter. In the root, shootward auxin transport depends on auxin transporter AUX1 and PIN2 [34]. Our work suggests the effect of ammonium on gravity response is mediated at least in part through repression of AUX1 and PIN2; the expression of both is prominently decreased by ammonium (Figure 6A and 6B).

Shootward auxin transport has been argued to regulate cell division as well as expansion [26]. Expressed in the lateral root cap, epidermis, and cortex, PIN2 is required for transporting auxin from the root tip to the elongation zone [15] and *pin2* mutants sometimes have lowered rates of cell production [26]. However, under our conditions, cell production rate of *eir1-1* was little different than that of wild type (Table 1), an observation that implies shootward auxin flux plays a secondary role in setting rates of cell production. Furthermore ammonium strongly reduced the expression of PIN2 (Figure 5B), which would be expected to limit the amount of auxin transported through the meristem, but ammonium reduced cell production rate in wild type and *eir1* alike (Table 1), again divorcing shootward auxin transport and cell production. A similar argument applies to AUX1, a molecule required for efficient auxin transport but in whose absence, cell production and elemental expansion are little affected, and ammonium remains able to regulate root growth.

In conclusion, primary root growth inhibited by physiological concentrations of ammonium supplied at the root tips is separated from shootward auxin transport mediated by AUX1 and PIN2, although ammonium suppresses AUX1 and PIN2 expression, which inhibits gravitropism. In parallel ammonium inhibits both division and expansion pathways, but the mechanisms behind these inhibitions remain to be elucidated.

## References

[1] Gazzarrini S, Lejay L, Gojon A, Ninnemann O, Frommer WB, et al (1999) Three functional transporters for constitutive, diurnally regulated, and starvation-induced uptake of ammonium into *Arabidopsis* roots. Plant Cell 11: 937–947. 10330477 10.1105/tpc.11.5.937 PMC144234

[2] Wolt JD (1994) *Soil Solution Chemistry*, John Wiley & Sons.

[3] Mosier A, Syers JK, Freney JR (2004) *Agriculture and the Nitrogen Cycle: Assessing the Impacts of Fertilizer Use on Food Production and the Environment*. Island Press, Washington, USA.

[4] Britto DT, Siddiqi MY, Glass AD, Kronzucker HJ (2001) Futile transmembrane NH_4_ ^+^ cycling: a cellular hypothesis to explain ammonium toxicity in plants. Proceedings of the National Academy of Sciences of the United States of America 98: 4255–4258. 11274450 10.1073/pnas.061034698 PMC31212

[5] Britto DT, Kronzucker HJ (2002) NH_4_ ^+^ toxicity in higher plants: a critical review. Journal of Plant Physiology 159: 567–584.

[6] Qin C, Qian W, Wang W, Wu Y, Yu C, et al (2008) GDP-mannose pyrophosphorylase is a genetic determinant of ammonium sensitivity in Arabidopsis thaliana. Proceedings of the National Academy of Sciences of the United States of America 105: 18308–18313. 19011088 10.1073/pnas.0806168105 PMC2587558

[7] Li Q, Li B, Kronzucker HJ, Shi W (2010) Root growth inhibition by NH_4_ ^+^ in *Arabidopsis* is mediated by the root tip and is linked to NH_4_ ^+^ efflux and GMPase activity. Plant, Cell & Environment 33: 1529–1542. 10.1111/j.1365-3040.2010.02162.x 20444215

[8] Rogato A, Apuzzo E, Barbulova A, Omrane S, Parlati A, et al (2010) Characterization of a developmental root response caused by external ammonium supply in *Lotus japonicus* . Plant Physiology 154: 784–795. 20688979 10.1104/pp.110.160309 PMC2948985

[9] Yuan L, Loqué D, Kojima S, Rauch S, Ishiyama K, et al (2007) The organization of highaffinity ammonium uptake in *Arabidopsis* roots depends on the spatial arrangement and biochemical properties of AMT1-type transporters. Plant Cell 19: 2636–2652. 17693533 10.1105/tpc.107.052134 PMC2002620

[10] Beemster GTS, Baskin TI (1998) Analysis of cell division and elongation underlying the developmental acceleration of root growth in *Arabidopsis thaliana* . Plant Physiology 116: 1515–1526. 9536070 10.1104/pp.116.4.1515 PMC35060

[11] Baskin TI (2012) Patterns of root growth acclimation: constant processes, changing boundaries. WIREs Dev Biol doi: 10.1002/wdev.94. 10.1002/wdev.94 23799631

[12] Overvoorde P, Fukaki H, Beeckman T (2010) Auxin control of root development. Cold Spring Harb Perspect Biol 2: a001537. 20516130 10.1101/cshperspect.a001537 PMC2869515

[13] Friml J, Benková E, Blilou I, Wisniewska J, Hamann T, et al (2002a) AtPIN4 mediates sink driven auxin gradients and root patterning in *Arabidopsis* . Cell 108: 661–673. 11893337 10.1016/s0092-8674(02)00656-6

[14] Friml J, Wisniewska J, Benková E, Mendgen K, Palme K (2002b) Lateral relocation of auxin efflux regulator PIN3 mediates tropism in *Arabidopsis* . Nature 415: 806–809. 11845211 10.1038/415806a

[15] Friml J (2003) Auxin transport - shaping the plant. Current Opinion in Plant Biology 6: 7–12. 12495745 10.1016/s1369526602000031

[16] Petrášek J, Friml J (2009) Auxin transport routes in plant development. Development 136: 2675–2688. 19633168 10.1242/dev.030353

[17] Cao Y, Class ADM, Crawford NM (1993) Ammonium inhibition of *Arabidopsis* root growth can be reversed by potassium and by auxin resistance mutations *aux1*, *axr1*, and *axr2* . Plant Physiology 102: 983–989. 8278539 10.1104/pp.102.3.983 PMC158872

[18] Kudoyarova GR, Farkhutdinov RG, Veselov SY (1997) Comparison of the effects of nitrate and ammonium forms of nitrogen on auxin content in roots and the growth of plants under different temperature conditions. Plant Growth of Regulation 23: 207–208.

[19] Zou N, Li B, Dong G, Kronzucker HJ, Shi W (2012) Ammonium-induced loss of root gravitropism is related to auxin distribution and TRH1 function, and is uncoupled from the inhibition of root elongation in *Arabidopsis* . Journal of Experimental Botany 63: 3777–3788. 22407650 10.1093/jxb/ers068

[20] Pickett FB, Wilson AK, Estelle M (1990) The aux1 mutation of *Arabidopsis* confers both auxin and ethylene resistance. Plant Physiology 94: 1462–1466. 16667854 10.1104/pp.94.3.1462 PMC1077399

[21] Roman G, Lubarsky B, Kieber JJ, Rothenberg M, Ecker JR (1995) Genetic analysis of ethylene signal transduction in *Arabidqsis thaliana*: five novel mutant loci integrated into a stress response pathway. Genetics 139: 1393–1409. 7768447 10.1093/genetics/139.3.1393 PMC1206465

[22] Colón-Carmona A, You R, Haimovitch-Gal T, Doerner P (1999) Spatio-temporal analysis of mitotic activity with a labile cyclin–GUS fusion protein. The Plant Journal 20: 503–508. 10607302 10.1046/j.1365-313x.1999.00620.x

[23] Geisler M, Jablonska B, Springer PS (2002) Enhancer trap expression patterns provide a novel teaching resource. Plant Physiology 130: 1747–1753. 12481057 10.1104/pp.011197 PMC1540270

[24] Marchant A, Kargul J, May ST, Muller P, Delbarre A, et al (1999) AUX1 regulates root gravitropism in *Arabidopsis* by facilitating auxin uptake within root apical tissues. EMBO Journal 18: 2066–2073. 10205161 10.1093/emboj/18.8.2066 PMC1171291

[25] Xu J, Scheres B (2005) Dissection of arabidopsis ADP-RIBOSYLATION FACTOR 1 function in epidermal cell polarity. Plant Cell 17: 525–536. 15659621 10.1105/tpc.104.028449 PMC548823

[26] Blilou I, Xu J, Wildwater M, Willemsen V, Paponov I, et al (2005) The PIN auxin efflux facilitator network controls growth and patterning in *Arabidopsis* roots. Nature 433: 39–44. 15635403 10.1038/nature03184

[27] Morris AK, Silk WK (1992) Use of a Flexible Logistic Function to Describe Axial Growth of Plants. Bulletin of Mathematical Biology 54: 1069–1081.

[28] Silk WK (1992) Steady form from changing cells. International Journal of Plant Sciences 153: 49–58.

[29] Dello Ioio R, Scaglia Linhares F, Scacchi E, Casamitjana-Martinez E, Heidstra R, et al (2007) Cytokinins determine arabidopsis root-meristem size by controlling cell differentiation. Current Biology 17: 678–682. 17363254 10.1016/j.cub.2007.02.047

[30] Malamy JE, Benfey PN (1997) Organization and cell differentiation in lateral root of *Arabidopsis thaliana* . Development 124: 33–44. 9006065 10.1242/dev.124.1.33

[31] Zhang H, Forde B (1998) An *Arabidopsis* MADS box gene that controls nutrient-induced changes in root architecture. Science 279: 407–409. 9430595 10.1126/science.279.5349.407

[32] Remans T, Nacry P, Pervent M, Filleur S, Diatloff E, et al (2006) The *Arabidopsis* NRT1.1 transporter participates in the signaling pathway triggering root colonization of nitrate-rich patches. Proceedings of the National Academy of Sciences 103: 19206–19211. 10.1073/pnas.0605275103 PMC1748200 17148611

[33] Jiang K, Feldman LJ (2005) Regulation of root apical meristem development. Annual Review of Cell and Developmental Biology 21: 485–509. 10.1146/annurev.cellbio.21.122303.114753 16212504

[34] Swarup R, Kramer E, Perry P, Knox K, Leyser O, et al (2005) Root gravitropism requires lateral root cap and epidermal cells for transport and response to a mobile auxin signal. Nature Cell biology 7: 1057–1065. 16244669 10.1038/ncb1316

[35] Robert HS, Friml J (2009) Auxin and other signals on the move in plants. Nucleic Acids Research 5: 325–332. 10.1038/nchembio.170 19377459

[36] Michniewicz M, Zago MK, Abas L, Weijers D, Schweighofer A, et al (2007) Antagonistic regulation of PIN phosphorylation by PP2A and PINOID directs auxin flux. Cell 130: 1044–1056. 17889649 10.1016/j.cell.2007.07.033

[37] Lewis DR, Miller ND, Splitt BL, Wu GS, Spalding EP (2007) Separating the Roles of Acropetal and Basipetal Auxin Transport on Gravitropism with Mutations in Two *Arabidopsis Multidrug Resistance-Like* ABC Transporter Genes. Plant Cell 19: 1838–1850. 17557805 10.1105/tpc.107.051599 PMC1955737

[38] Walch-Liu P, Neumann G, Bangerth F, Engels C (2000) Rapid effects of nitrogen form on leaf morphogenesis in tobacco. Journal of Experimental Botany 51: 227–237. 10938829 10.1093/jexbot/51.343.227

[39] West G, Inzé D, Beemster GTS (2004) Cell cycle modulation in the response of the primary root of Arabidopsis to salt stress. Plant Physiology 135: 1050–1058. 15181207 10.1104/pp.104.040022 PMC514139

[40] Sharp RE, Silk WK, Hsiao TC (1988) Growth of the maize primary root at low water potentials I. Spatial distribution of expansive growth. Plant Physiology 87: 50–57. 16666126 10.1104/pp.87.1.50 PMC1054698

[41] Yamaguchi M, Valliyodan B, Zhang J, Lenoble ME, Yu O, et al (2010) Regulation of growth response to water stress in the soybean primary root. I. Proteomic analysis reveals region-specific regulation of phenylpropanoid metabolism and control of free iron in the elongation zone. Plant, Cell and Environment 33: 223–243. 10.1111/j.1365-3040.2009.02073.x 19906149

[42] Ma Z, Baskin TI, Brown KM, Lynch JP (2003) Regulation of root elongation under phosphorus stress involves changes in ethylene responsiveness. Plant Physiology 131: 1381–1390. 12644687 10.1104/pp.012161 PMC166897

[43] Pérez-Torres C, López-Bucio J, Cruz-Ramírez A, Ibarra-Laclette E, Dharmasiri S, et al (2008) Phosphate availability alters lateral root development in *Arabidopsis* by modulating auxin sensitivity via a mechanism involving the TIR1 auxin receptor. Plant Cell 20: 3258–3272. 19106375 10.1105/tpc.108.058719 PMC2630440

[44] Giehl R, Lima J, von Wirén N (2012) Localized iron supply triggers lateral root elongation in *Arabidopsis* by altering the AUX1-mediated auxin distribution. Plant Cell 24: 33–49. 22234997 10.1105/tpc.111.092973 PMC3289578

